# Sexually-dimorphic targeting of functionally-related genes in COPD

**DOI:** 10.1186/s12918-014-0118-y

**Published:** 2014-11-28

**Authors:** Kimberly Glass, John Quackenbush, Edwin K Silverman, Bartolome Celli, Stephen I Rennard, Guo-Cheng Yuan, Dawn L DeMeo

**Affiliations:** Department of Biostatistics and Computational Biology, Dana-Farber Cancer Institute, Boston, MA USA; Department of Biostatistics, Harvard School of Public Health, Boston, MA USA; Channing Division of Network Medicine, Brigham and Women’s Hospital and Harvard Medical School, Boston, MA USA; Division of Pulmonary and Critical Care Medicine, Brigham and Women’s Hospital, Boston, MA USA; Division of Pulmonary, Critical Care, Sleep and Allergy, University of Nebraska Medical Center, Omaha, NE USA

**Keywords:** Network modeling, Gene regulation, Regulatory networks, Sexual-dimorphism, Chronic Obstructive Lung Disease

## Abstract

**Background:**

There is growing evidence that many diseases develop, progress, and respond to therapy differently in men and women. This variability may manifest as a result of sex-specific structures in gene regulatory networks that influence how those networks operate. However, there are few methods to identify and characterize differences in network structure, slowing progress in understanding mechanisms driving sexual dimorphism.

**Results:**

Here we apply an integrative network inference method, PANDA (*P*assing *A*ttributes between *N*etworks for *D*ata *A*ssimilation), to model sex-specific networks in blood and sputum samples from subjects with Chronic Obstructive Pulmonary Disease (COPD). We used a jack-knifing approach to build an ensemble of likely networks for each sex. By adapting statistical methods to compare these network ensembles, we were able to identify strong differential-targeting patterns associated with functionally-related sets of genes, including those involved in mitochondrial function and energy metabolism. Network analysis also identified several potential sex- and disease-specific transcriptional regulators of these pathways.

**Conclusions:**

Network analysis yielded insight into potential mechanisms driving sexual dimorphism in COPD that were not evident from gene expression analysis alone. We believe our ensemble approach to network analysis provides a principled way to capture sex-specific regulatory relationships and could be applied to identify differences in gene regulatory patterns in a wide variety of diseases and contexts.

**Electronic supplementary material:**

The online version of this article (doi:10.1186/s12918-014-0118-y) contains supplementary material, which is available to authorized users.

## Background

Chronic respiratory diseases, including Chronic Obstructive Pulmonary Disease (COPD), are among the most likely causes of death in the United States; COPD ranks third only after heart disease and all forms of cancer combined [1]. In the past COPD was thought to primarily affect males, but in recent years the number of females with COPD has greatly increased, and currently more women die of COPD than men [2]. Some of the changing epidemiology is likely due to an increase in female cigarette use during the 1960s. However, current research also suggests biological causes for the apparent sexual-dimorphism in the disease, with women having a higher susceptibility [3-5], an overall more severe COPD course even with the same level of tobacco exposure [6], and an increase in severe symptoms at a younger age [2,7].

Investigating sex differences in disease is a critical area of investigation [8,9] and a wide number of diseases are known to effect men and women differently [10]. It has been noted that many sexually dimorphic features are likely not primarily due to genetic variation [11]. On the other hand, network-modeling of transcriptomes in model organisms has demonstrated sexually dimorphic higher-order gene interactions [12]. Consequently, systems-based approaches have great potential for exploring sex-differences in human traits [13,14]. In this study we leverage gene expression data from subjects with COPD to build sex-specific networks and investigate whether alterations in gene regulation might contribute to sexual-dimorphism in COPD. The methods described here are not limited to analysis of lung disease but are generalizable to other diseases that demonstrate sexually dimorphic characteristics.

Gene regulation involves the concerted activity of many distinct but non-independent regulatory mechanisms [12,14]. While no single experimental assay can fully capture the complexity of a given biological system, each provides information concerning a particular feature that influences, or results from, the state of a cell. Because of the complexity of gene regulatory processes, there is increased interest in modeling approaches capable of integrating multiple sources of regulatory information [15-19], and evidence suggests that these methods perform much better than those using individual data types in isolation [20].

Along these lines, we developed PANDA (*P*assing *A*ttributes between *N*etworks for *D*ata *A*ssimilation) [21], a “message passing” network inference method that integrates multiple types of genomic data. PANDA models information flow through networks under the assumption that both “transmitters” and “receivers” play active roles in modulating regulatory processes. In PANDA’s model of gene regulatory control, transcription factors are the transmitters and the receivers are their target genes. A set of initial connections linking transcription factors to potential downstream targets is inferred by mapping transcription factor binding sites (TFBS) to the genome. Gene expression profiles provide information on shared activation states for elements in the network and protein-protein interaction data provide information on co-regulatory processes. PANDA starts with initial networks and then uses the various data to iteratively update the network structures to more accurately fit the available information, until the process converges on a consensus regulatory network.

In applying PANDA, we construct phenotype-specific models and then look for variation in TF-target interactions (“edges”) to explore regulatory differences. One surprising result of applying PANDA in such a comparative analysis is that we are able to observe meaningful changes in regulatory patterns even for genes that are not differentially expressed [22].

The comparative analysis of phenotype-specific networks enabled by PANDA makes it particularly useful for studying sexual dimorphism in health and disease, where the absolute levels of gene expression in disease may be similar in male and female tissues but in which different regulatory processes may be active [14], including differences in transcription factor regulation in the presence of sex hormones [23,24]. If this is the case, identifying sexually dimorphic network variability and associating these network characteristics with specific disease processes can lead not only to a better understanding of the disease, but also to therapies optimized for men and women.

In this study we begin by analyzing blood and sputum gene expression data from subjects with COPD. We then explore whether gene regulatory networks, estimated using these data, contain sex-specific regulatory patterns. To do this we use PANDA to model “ensembles” of sex-specific regulatory networks in COPD and use these network ensembles to identify differences in network topologies that are associated with biological functions in a sex-specific manner. As opposed to analyzing or contrasting the properties of single networks, this ensemble approach to network analysis allows for the statistical quantification of network features. In this application, we demonstrate how Gene Set Enrichment Analysis (GSEA), which was originally designed to quantify the association of gene sets with differential expression changes, can be used to estimate the association of gene sets with alterations in network features in light of this ensemble approach. However, more generally, our ensemble approach to network modeling allows for the principled investigation of differences in network properties using statistical tools developed for genomic and other high-dimensional data.

## Results and discussion

### Genes and gene sets are not strongly differentially-expressed between males and females with COPD in either blood or sputum

**Figure 1:**
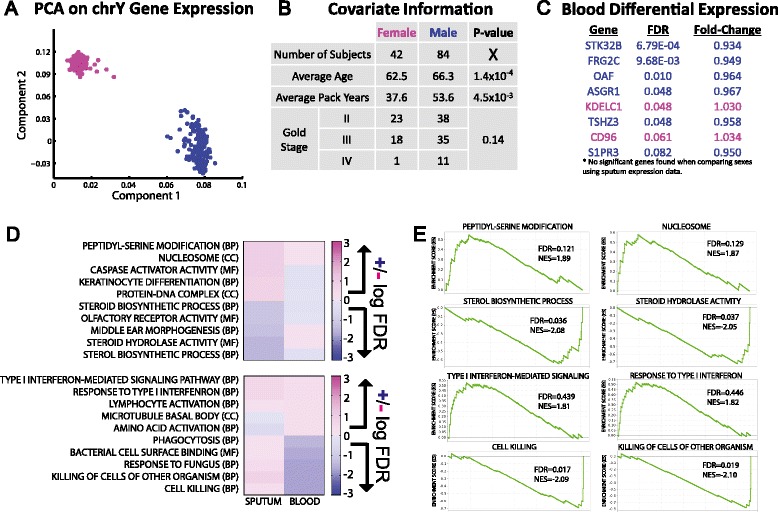
**Comparison of males and females with COPD using a standard differential-expression analysis approach. (A)** A PCA analysis on expression data using genes located on the Y chromosome. Males and females cluster into two groups. **(B)** Covariate information for the 42 female and 84 male subjects included in the analysis. The statistical difference between sexes for age and pack-years was calculated using an unpaired two-sample t-test and the statistical difference between the sexes for GOLD stage was calculated by applying a chi-squared test to a two by three (sex by stage) contingency table. **(C)** The top most differentially-expressed genes based on a using an unpaired two-tailed t-test (after specifically excluding genes those on the sex chromosomes). Genes with higher average expression in female are colored pink and those with higher average expression in male are colored blue. **(D)** The results of a GSEA analysis looking for GO category differential-expression between males and females. The five most differentially-expressed GO categories in males and females in either sputum or blood are shown. Deeper shades of pink are used to denote greater significance in female while deeper shades of blue indicate greater significance in males. The scale is based on the –log FDR significance for categories enriched in females, resulting in positive values, and on the + log FDR significance for categories enriched in males, resulting in negative values. Note that the color-range extends to an FDR significance of 10^−3^ in each sex even though the most significant categories found in this analysis only reach an FDR significance of around 10^−2^. **(E)** GSEA “enrichment plots” for the two most significantly differentially-expressed GO categories according to the GSEA analysis in males and females in either sputum or blood.

We obtained and analyzed gene expression data in sputum and blood samples from 132 subjects (44 females and 88 males) with COPD enrolled in the ECLIPSE study [25]. Affymetrix CEL files were downloaded and normalized using RMA [26], with probe-sets mapped to Entrez-gene IDs using a custom CDF [27]. An initial quality control of this data was performed by running a principal component analysis on the expression values for the 24 probe-sets located on the Y chromosome. A plot of the first versus the second principal component (Additional file 1: Figure S1A) indicates that although most samples cluster according to the sex ascribed in the phenotype data, there are six samples which do not cluster as expected. To minimize potential noise due to poor quality data or sex misclassification, we eliminated these six subjects from further consideration, leaving 42 female and 84 male COPD subjects with both sputum and blood gene expression data. A principal component analysis plot for these remaining samples, generated using expression information for genes located on the Y chromosome, is shown in Figure 1A; age, COPD Global Initiative for Chronic Obstructive Lung Disease (GOLD) stage based on spirometry and pack-years of cigarette smoking for the corresponding subjects are shown in Figure 1B. We compared the age, COPD GOLD stage and pack-years of cigarette smoking between men and women and observe significant differences in age and pack-years but no significant difference in disease stage. This is consistent with previous observations that women often get similarly severe COPD at a younger age and with less smoke exposure [2,6,7] and highlights the importance of understanding the biologic features mediating sexual dimorphism in COPD. All subjects included in this analysis are former smokers.

For the remaining 126 subjects, a genome-wide differential expression analysis including the sex chromosome genes serves as a strong positive control on the expression data as the results identify many expected sex-related differences (Additional files 1: Figure S2 and Additional file 1: Tables S1–S2).We next excluded genes on the sex chromosomes and tested if autosomal genes were strongly differentially-expressed between males and females in either the sputum or blood samples, using an unpaired two-sample t-test. Using the sputum samples, no genes are significantly differentially expressed between males and females at an FDR less than 0.1. Only eight autosomal genes (listed in Figure 1C) are significantly differentially-expressed in blood between female and male COPD subjects at an FDR threshold of 0.1, suggesting that the removal of sex chromosome genes largely mitigates the sex-specific gene expression signal. Consequently, subsequent analyses exclude genes on the sex chromosomes.

Although very few autosomal *genes* are significantly differentially-expressed when comparing samples from males and females, it is still possible that *groups* of interacting genes, representing particular biological functions, might be *collectively* differentially-expressed in a sex-specific manner. We evaluated this possibility by performing Gene Set Enrichment Analysis (GSEA) [28]. We downloaded the java implementation of GSEA (http://www.broadinstitute.org/gsea/) and tested for the collective sex-specific differential expression for sets of genes annotated to Gene Ontology (GO) functional categories. GSEA uses a gene-by-sample table of expression values and information concerning sample features (in this analysis, subject sex) to rank genes based on their differential expression. It then uses this ranking to test if sets of genes (for example, those annotated to a particular GO term) have consistent changes in expression patterns, in our case, consistently higher expression levels in one sex compared to the other.

Figure 1D shows the five most differentially-expressed functional gene sets (hereafter, simply “functions” or “GO terms”) in males and females for both sputum (top panel) and blood (bottom panel). Several of the corresponding GSEA enrichment plots are presented in Figure 1E. Although the top functions are only marginally significant, both the blood and sputum analysis includes several interesting results. In sputum, the most differentially-expressed functions reach an FDR significance in the range of 0.01 to 0.15 and include GO terms such as “sterol biosynthetic process” and “steroid hydrolase activity”, which may play a role in sexual dimorphism. The GO functions more highly expressed in COPD blood samples in males compared to females include “cell killing” and “phagocytosis”, processes potentially related to COPD pathogenesis and severity [29,30].

### Jack-knifing can be used to robustly estimate and compare regulatory networks

We also used a two-sample f-test to evaluate if the *variance* of any of the autosomal genes’ expression levels was significantly difference between females and males. We observe that in sputum samples over 1000 genes are differentially-variable at an FDR less than 0.1. We include these genes in Additional file 2. This observation, together with the plausible functional enrichment results, led us to next hypothesize that the differential *targeting* of biological functions may play a critical role in sexual dimorphism in COPD. Specifically, it is possible that genes are differentially *co-expressed*, even if their overall average expression levels are not significantly different. If this differential co-expression is taken as evidence of differential co-regulation, as is done in PANDA, then potential transcription factors that are differential-targeting these genes can be identified (Additional file 1: Figure S3).

**Figure 2:**
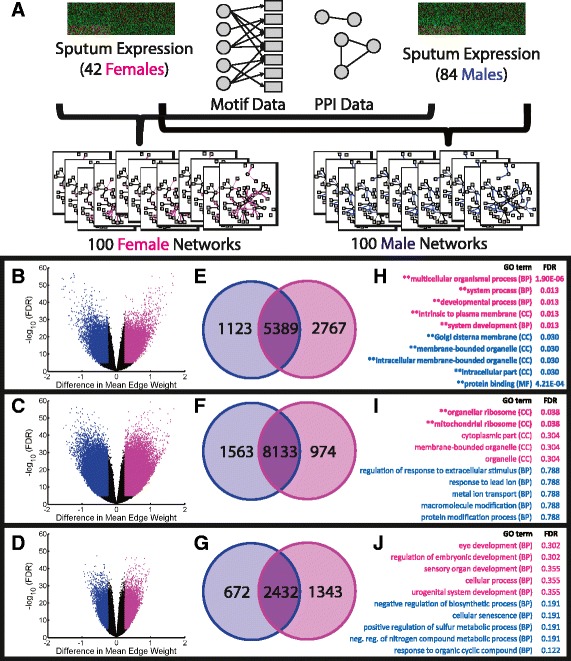
**Using ensembles of networks to robustly identify sex-specific interactions and their associated genes. (A)** A cartoon summary of how we use PANDA to build ensembles of networks using a jack-knifing approach to resample the original expression data multiple types. **(B-D)** Volcano plots of the difference in mean edge weight across two ensembles of networks compared to the p-value of the difference in the edge weight distributions in the two ensembles. Comparisons include **(B)** female versus male sputum networks, **(C)** female versus male blood networks, **(D)** female versus male “random” networks. Edges identified as “different” in each comparison are shown as either pink (female-specific) or blue (male-specific). **(E-G)** Venn diagrams showing the overlap in genes targeted by the female-specific (pink) or male-specific (blue) edges. Note that a gene can be targeted by both a male-specific and a female-specific edge, but by different upstream transcription factors. There is a high level of overlap in the genes targeted by the identified sex-specific edges in both the sputum and blood networks. **(H-J)** A hypergeometric probability was used to determine the significance of overlap in male-specific genes with genes annotated to GO categories, and female-specific genes with genes annotated to GO categories. The top five categories enriched in the males and females for each comparison are shown.

It has been suggested that regulatory relationships between transcription factors and genes likely have both stochastic and deterministic components, and thus may be better modeled by probability distributions as opposed to simple Boolean relationships [31,32]. Furthermore, in this application we recognized that differences in sample size between males and females could potentially influence predictions of regulatory network interactions. Motivated by this, we used PANDA [21] to calculate *ensembles* of networks based on jack-knifed sets of samples drawn from our initial male and female subject populations (Figure 2A).

Specially, As an input to PANDA, we constructed transcription-factor target networks using position-weight-matrices for 130 TFs recorded in the Jaspar database [33], mapping these to the promoter regions, defined as [−750,+250] base-pairs around the transcription start site. We also include information regarding physical protein-protein interactions between human transcription factors [34]. To build ensembles of networks, we used a “jack-knife” [35], randomly selecting ten samples without replacement to create 400 gene expression data sets, 100 for each of four sample sets (blood-female, blood-male, sputum-female, sputum-male). We then used PANDA to infer networks for each expression data set. As a negative control, we also created a version of the sputum expression data with a permutation of gene labels, and built sex-specific ensembles of networks for this randomized data.

This jack-knifing approach ensures that the predicted network edges are not strongly influenced by any one subject, as each network in our ensembles represents an estimate of the cellular regulatory network for a subset of the relevant samples. It also helps us regularize differences in sample size between the sexes as each of the reconstructed networks contains information from the same number of subjects. Further, our male and female ensembles each include one hundred networks, giving us the power to quantify the statistical properties of the estimated regulatory edges, something that would have been difficult or impossible had we simply estimated a single network for each sex and tissue-type combination. Although the jack-knifing approach does not allow us to directly model covariates (for example, differences in COPD severity or smoking histories), it helps mitigate their effect on the network predictions by modeling a distribution of networks, which are, on average, representative of the population, but whose variance likely represents the contribution of other factors.

We used an un-paired two-sample t-test to quantify differences in the distributions of predicted edge-weights between the sex-specific network ensembles. We also averaged the predicted edge weight across the networks in each ensemble, excluded edges with low average weights (<0) and, for the remaining edges, determined the difference in these average edge weight values between the ensembles. Figure 2B-D shows volcano plots of the difference in the average of each edge’s weight between the ensembles being compared, versus the FDR significance in the difference of edge weight distributions based on the t-test. We immediately observe that edge differences in the “random” volcano plot are not nearly as strong as those in the sputum and blood volcano plots; however, there are some differences, including edges that are “significantly” different according to the t-test. Consequently in this following network edge analysis we use a more stringent FDR cutoff than we did with the gene expression analysis.

We used a combination of the difference (absolute value >0.25), significance based on the t-test (FDR <10^−5^) and average edge weight (>0) to select differentially-called edges for each ensemble comparison. Female- and male-specific edges are shown in pink and blue, respectively, in Figures 2B-D. These criteria were chosen such that each sex-specific subnetwork contains edges that are both likely to be real (based on a positive edge weight) as well as different, both at an absolute and at a statistical level. The cutoff values themselves were selected such that each subnetwork contains between one and five percent of all possible edges, which may be close to an expected network density. We applied these same cutoffs to the “random” volcano in order to quantify the level of false positives in the differential subnetwork edge calls. Although there are likely false-positive edges in our identified subnetworks, for the selected cut-offs there are approximately 2.4 and 9.4 times more differentially-called edges in the sputum and blood volcanos compared to the random volcano, respectively. We note that this randomization control also illustrates that statistical differences calculated by contrasting various network properties should be viewed primarily as a rank-ordering as opposed to a true significance level.

We determined the genes targeted by these sex-specific edges and present the results as Venn diagrams (Figure 2E-G). Many genes (5389 in sputum and 8133 in blood) are targeted in both male and female subnetworks, although the network models indicate the regulation is governed by different transcription factors. This may partially explain why we previously observed only minimal differential gene expression patterns between the sexes; our network results suggest that although genes may be similarly expressed in both sexes, this is mediated by a distinct set of transcriptional regulators.

To assess whether the genes targeted in only one sex-specific subnetwork and not the other might be associated with specific biological functions, we used Fisher’s exact test to evaluate the enrichment of GO categories in these genes and observe some functional enrichment (Figure 2H-J). The signal appears to be strongest for the genes uniquely targeted in a sex-specific manner in the sputum-derived networks (Figure 2H); the sputum samples may be biologically “closer” to the disease as a lung source sample and may represent cellular process most likely to be associated with COPD.

### Network ensembles uncover differential-targeting patterns in men and women with COPD

We recognize that there are significant limitations to studying functional enrichment in a context that relies upon somewhat arbitrary thresholds in order to define differential subnetworks (Figure 2B-J). Firstly, this type of approach can be sensitive to the cutoffs used, opening the opportunity for potentially biased results when not used with caution. Additionally, selecting genes based on whether they are or are not targeted in a pair of networks ignores any *relative* level of differential targeting. Specifically, we observe a high level of overlap in target genes when comparing male and female subnetworks (see Figure 2E-G); however, there are multiple instances when a gene is targeted by many transcription factors in one subnetwork but by a much smaller number, or even a single TF in the other. Although we excluded these commonly targeted genes in the analysis shown in Figure 2E-J, one could imagine they might play a significant role in sex-specific differences in COPD.

Motivated to overcome these limitations, we next used the ensembles of networks generated by PANDA in a manner analogous to how we used the expression data to evaluate differential-enrichment of GO functions between the sexes. We previously observed that some sets of functionally-related genes are weakly differentially-expressed (Figure 1D); here we wish to address a similar, but distinctly different question within the network context. Namely, are sets of functionally-related genes differentially-*targeted*? In other words, do a set of functionally-related genes tend to have an increase (or decrease) in regulatory targeting in one sex-specific regulatory network context compared to another?

**Figure 3:**
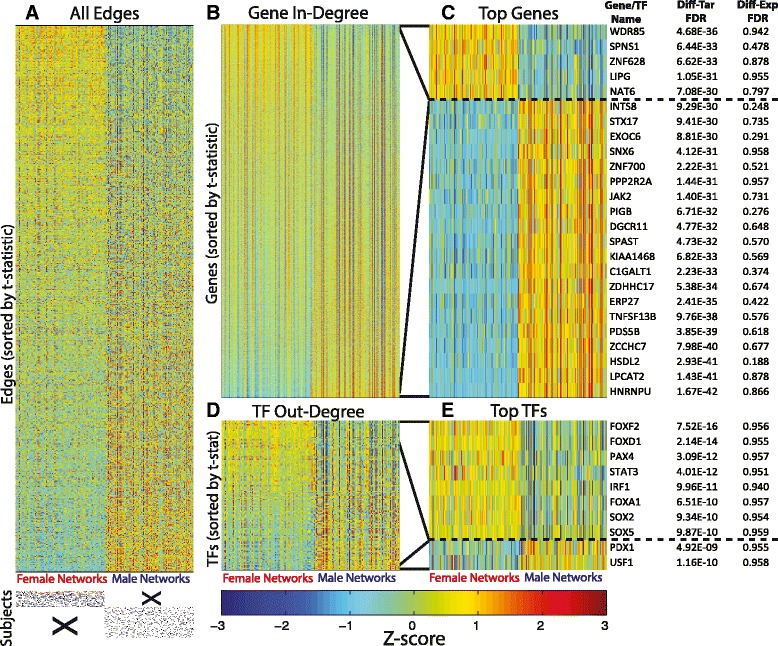
**Visualization of edge weight and the in- and out-degree of genes and TFs in ensembles of sputum networks. (A)** Edge weights for every possible transcription factor to gene interaction, where each row represents an edge, and each column represents one of the networks produced in the jack-knifing approach. Rows are ordered based on the t-statistic comparing the edge weight values and each row is Z-score normalized for visualization purposes only. **(B)** The in-degree, defined as the sum of all incoming edge weights, for each gene in the PANDA network reconstruction. Genes (rows) are ordered based on the t-statistic comparing the gene in-degree distributions in the two ensembles of networks (columns). Again, rows are Z-score normalized only for visualization purposes. **(C)** The twenty-five most differentially-targeted genes, identified as having the most significant difference in in-degree in the male compared to the female ensemble of networks. Both the significance of the differential-targeting and the level of differential-expression is shown. **(D)** The out-degree, defined as the sum of all outgoing edges, for each transcription factor in the reconstructed networks. Rows represent transcription factors and are again ordered based on the t-statistic comparing the distribution of in-degree values of the transcription factor in the two ensembles of networks. **(E)** The ten most differentially-targeting transcription factors, and their level of differential-expression. The majority of the differentially-targeted genes and differential-targeting transcription factors are not differentially-expressed.

In this analysis, instead of sets of *expression samples* associated with disease state and sex, we have sets of *regulatory networks*. Specifically, we have one hundred corresponding representative networks for each set of expression samples, and therefore one hundred predicted scores for each edge in those networks. Figure 3A shows a heat map of those scores for the male and female sputum networks. Some edges have consistently higher predicted edge weights in the male networks while others have consistently higher predicted edge weights in the female networks. We would like to relate these differences in network structure to differences in the regulation of biological functions.

To begin to address this question, within each of our sex-specific PANDA predicted networks, we assigned every gene a score based on its “in-degree”, which is defined as the sum of the weights of all edges pointing to that gene. Figure 3B shows the in-degree values side-by-side for the male and female sputum networks. We sorted genes in this figure based on the statistical difference in the in-degree values between the two network ensembles, as measured by an unpaired two-sample t-test. As with the edges, we observe that some genes are consistently much more highly targeted in the male networks, while others are consistently much more highly targeted in the female networks. The twenty-five most differentially-targeted genes, based on the t-test comparison, are shown in Figure 3C. As a control for this analysis we also reconstructed one hundred networks built after permuting the sex-labels of the subjects (Additional file 1: Figure S4A). We observe that the differential-targeting observed for these genes is much greater than expected by chance.

Our calculated in-degree values give an indication of how heavily a gene is targeted in a given network. Edge-weights predicted by PANDA correspond to how likely a given regulatory interaction is to exist and edges that represent either activating or repressing interactions can have similarly high weights. Consequently, genes with relatively higher degrees are not necessarily “more activated,” they may in fact be repressed (if they are highly targeted by more repressors than activators), or neither (if they are equally targeted by both activators and repressors). Therefore, a change in a gene’s degree between two sets of networks is not necessarily related to either an increase or decrease in its expression level, but instead suggests changes in its regulatory control. Consistent with this framework, even the most strongly differentially-targeted genes do not appear to be strongly differentially-expressed (Figure 3C). We therefore suggest that these differences in gene targeting likely represent a sexually-dimorphic disease-related re-wiring of the cellular network and that understanding the biological implications of these structural changes may provide insight into the mechanisms driving disease morphology and lead to suggestions for sex-specific therapies.

We also calculated the “out-degree” of TFs in these networks, or the sum of the weights of all edges pointing from a TF, and show the results in Figure 3D-E. As before, we observe strong sex-specific differences in targeting patterns, even though the TFs themselves are not differentially-expressed. These results suggest that differences in regulatory patterns in the absence of strong differential expression exist around the regulating TFs as well as the regulated genes. Thus the sex differences we observe appear to be strongest at the level of the network “edge” and not necessarily in the individual “node” (gene and TF) states.

### Biological functions are strongly associated with sexually-dimorphic targeting in COPD subjects

Our analysis suggests that although there is little difference in gene expression levels between males and females with COPD in either blood or sputum, there are likely different regulatory mechanisms associated with and potentially mediating the disease state. If this is true, one would expect that alterations in network structure should be concentrated around genes representing particular functional classes representing changes in the *mechanisms of activation*, rather than downstream changes in gene expression. Therefore, next we sought to identify sexually dimorphic *differentially-targeted* functions. We created “gene-by-network” tables for each ensemble of networks, where the values are the in-degrees of the genes (the level of targeting identified by PANDA) in each of our predicted networks. We then ran GSEA using these in-degree values instead of expression to evaluate if functionally-related sets of genes gain or lose targeting.

**Figure 4:**
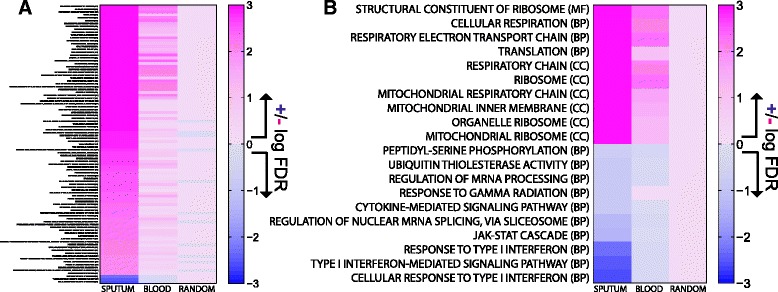
**Sexually-dimorphic targeting of biological functions in Sputum and Blood networks. (A)** All GO categories significantly differentially-targeted (FDR < 0.01) using a GSEA-type approach to compare gene targeting in male and female networks derived from either sputum or blood expression data. Many functional categories have genes that appear to be much more highly targeted in the female networks compared to the male networks. There is a high level of agreement between the differential-targeted GO categories in both the sputum and blood networks, but the enrichment disappears in the “random” networks. **(B)** The ten most differentially-targeted pathways enriched in the female and the male sputum networks.

Running GSEA on differential gene-degree leads to some striking results (Figure 4A). First, despite the lack of strong differential-*expression* noted previously, directly comparing male versus female *networks* using this enrichment method reveals strong patterns of differential-*targeting*, with many functions that have significantly (FDR < 0.01) more targeting in the female compared to the male networks (Figure 4A). Differential-targeting of these functional categories is absent in networks reconstructed after permuting the sex-labels (Additional file 1: Figure S4B). Furthermore, the results are highly consistent when comparing female and male networks built using either the sputum or blood samples (although there is overall greater enrichment for differential-targeting of functions in the sputum). In contrast, repeating the analysis using networks constructed from “random” expression data shows no strong differential-targeting patterns.

Closer inspection of the differentially-targeted functions shows many to be highly-related based on their biological role and gene content. Figure 4B shows the ten most differentially-targeted functions in females and males in sputum. A closer inspection of the expression levels of the genes annotated to these top functional categories shows that they appear to be associated with disease stage (Additional file 1: Figure S5), supporting their relevance to COPD. The pathways most significantly targeted in men are related to type I interferon, which has previously been implicated in the sexual dimorphism in response to viral infections (drivers of COPD exacerbations) [36,37] and in autoimmune diseases [38]. They are also consistent with previous observations that immune functions are enriched in male COPD-associated genes [39]. The pathways more highly targeted in women are all related in some way to mitochondrial function, which has previously been implicated in the modulation and development of lung disease [40,41]. Cigarette smoking has also been shown to change mitochondrial morphology [42] and abnormal mitochondrial function is described in patients with COPD [43,44].

Because of its maternal inheritance [45,46], the mitochondria has long been associated with sex-differences. Sex hormones play an important role in controlling mitochondrial biogenesis and activities [47-50]. In neuronal cells ER-beta is localized in the mitochondria and mediates mitochondrial vulnerability to oxidative damage [51,52]; it also impairs mitochondrial oxidative metabolism in mesothelioma [53]. Interestingly, estrogen receptors are reduced in the mitochondria of epithelial cells from asthmatic lungs [54]. In addition, multiple peroxisome proliferator-activated receptors (PPARs), a class of nuclear hormone receptor proteins, have lower expression levels in COPD patients. This activity corresponds to lower expression levels of the PPAR-γ co-activator PGC-1α [55], a key regulator of energy metabolism [56] and an inducer of mitochondria biogenesis [57]. Thus differential-targeting of mitochondrial functions is consistent both with known biology concerning sexual-dimorphism and COPD.

We have performed two analyses to confirm that the strong differential-targeting of biological functions we observe in these networks is not a consequence of our specific approach. First, we repeated the ensemble network reconstruction on the sputum expression data, but modified our sampling technique to match covariates between each selected set of ten female and ten male samples; the conclusions of this covariate-matched analysis are nearly identical to what we observe with the random sampling (Additional file 1: Figure S6). Secondly, we ran (1) one hundred GSEA differential-expression analyses, one for each set of ten versus ten expression samples, and (2) one hundred GSEA differential-targeting analyses, one for each female versus male network reconstructed from these samples. Across these analyses we again observe consistently strong differential-targeting of many biological functions (Additional file 1: Figure S7).

### Transcription factors mediate differential-targeting patterns in COPD

**Figure 5:**
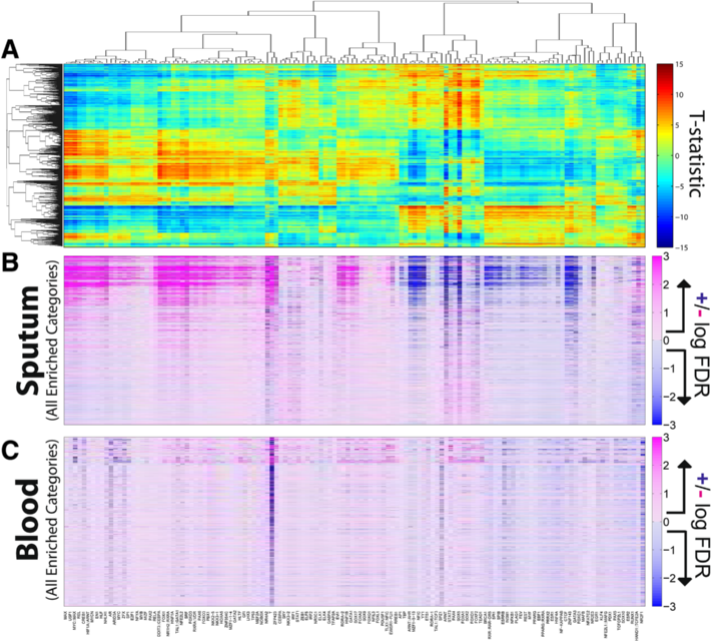
**Transcription-factor differential-targeting of biological functions. (A)** A hierarchical clustering of the t-statistic associated with differential edge weight between ensembles of female and male sputum networks. Each point in the matrix represents the t-statistic of an individual edge extending from a transcription factor (column) to a gene (row) **(B-C)** The statistical enrichment of GO categories in genes differentially-targeted by a transcription factor between male and female **(B)** sputum and **(C)** blood networks. All categories significantly (FDR < 0.01) differentially-targeted by at least one of the transcription factors, in the given tissue-type, is shown. The columns (transcription factors) are ordered identically to the hierarchical clustering in **(A)** and we observe a strong correlation with the transcription-factor differential-targeting of GO categories in **(B)**. Although there is some similarity in the transcription-factor differential-targeting of these functional sets of genes in the sputum and blood networks, there is overall less enrichment in the blood comparison.

To gain a better appreciation for the network-level patterns that might be driving the identified functional alterations, we constructed a gene-by-TF matrix of the t-statistic values associated with the differences in edge weights predicted for the female compared to the male sputum networks and performed a complete-linkage hierarchical clustering using a Pearson correlation coefficient distance (Figure 5A). The resulting heatmap, where the rows are genes and the columns are transcription factors, shows clear patterns involving sets of transcription factors differentially-targeting sets of genes in the female and male networks. Given these results, we next sought to identify if particular transcription factors might be mediating the differential-targeting of biological functions between men and women.

For each jack-knife iteration PANDA calculates an edge weight for every possible transcription factor to gene interaction representing the likelihood that the TF regulates that target gene. We used this information to design TF-specific gene-by-network tables. We ran GSEA on these TF-specific tables to evaluate if any functions are more strongly targeted by an individual TF in one of our ensembles of networks compared to the other. The results of the female versus male comparison in both sputum and blood are shown in Figure 5B-C, with the transcription factors shown in the same order as in the hierarchical clustering and each row representing a biological function found to be enriched (FDR < 0.01) when contrasting at least one set of male or female TF-specific edges. We find more than 1000 GO functions differentially-targeted between the sexes by at least one transcription factor in sputum, and almost 900 in blood. As with the gene in-degree analysis we once again see much stronger differential-targeting of functions in the sputum network comparison relative to the blood network comparison.

### Disease-specific regulators of sexually-dimorphic functional targeting

**Figure 6:**
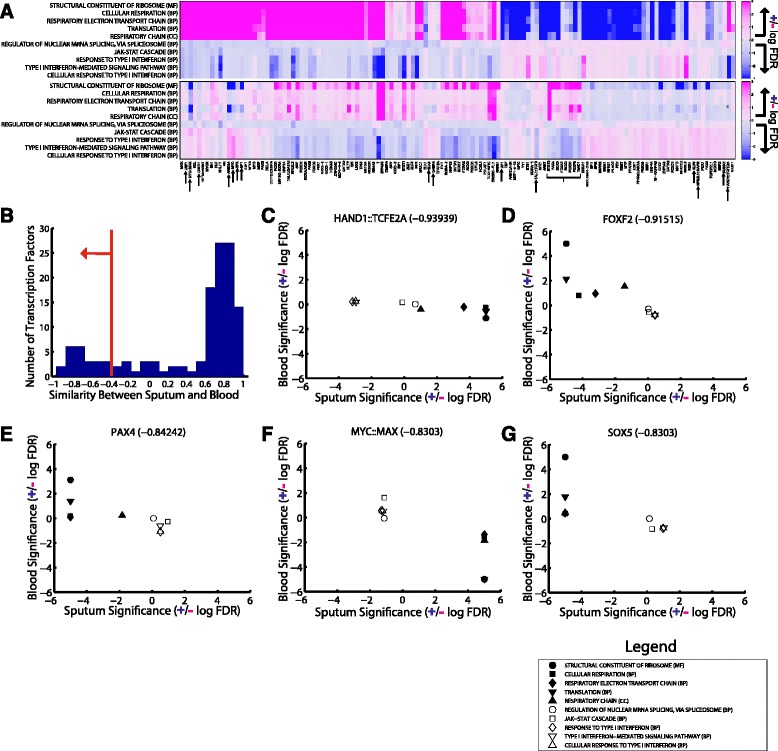
**Identifying disease-specific drivers of sexually-dimorphic functional targeting. (A)** Sputum (top panel) and blood (bottom panel) transcription-factor specific enrichment for differential-targeting of the top five GO functions identified in either males or females in Figure 4B. **(B)** A distribution of the similarity between differential-targeting patterns of transcription factors in sputum and blood network, as measured using the Spearman correlation. A red line indicates the cutoff used to identify transcription factors that have opposite sex-specific regulatory patterns in sputum compared to blood networks. **(C-G)** Plots comparing individual transcription factors’ sex-specific differential-targeting of these GO functions (five in female, filled shapes, and five in male, hollow shapes) in the sputum versus the blood networks.

In order to better interpret this information, we focused on our previously-identified ten most differentially targeted functions (see Figure 4B) and present the TF-specific GSEA results in Figure 6A. We see overall consistency between the blood and sputum sexually-dimorphic targeting of these functions by individual transcription factors. However, a handful of transcription factors appear to have opposite patterns in the sputum and the blood networks.

One limitation of directly comparing data from men and women with COPD is that without healthy controls it is unclear whether the systemic changes and high level of consistency we observe in the blood and sputum network analyses are important for sex-related differences in the disease or are a consequence of normal sex differences in cellular regulation. However, we reasoned that the sputum networks should be “closer” to lung disease, and thus transcription factors that are regulating biological functions in sputum but not in blood may be the most important drivers of sex-specific *and* disease-specific functional regulation. Therefore, to partially address our lack of healthy controls, we next directly compared the transcription-factor specific differential-targeting of functions in the sputum versus the blood networks.

We quantified differences in transcription-factor level targeting of the ten functions in Figure 6A by calculating, for each transcription factor, the Spearman correlation between the significance levels in the sputum sex-specific network comparison and the significance levels in the blood sex-specific network comparison. A distribution of these correlation values is shown in Figure 6B.

Most transcription factors have a high positive correlation value, indicating that they are increasing/decreasing their targeting of these biological functions between men and women similarly in both sputum and blood networks. Some of this sexually-dimorphic targeting may be related to COPD, however, it is also possible, since this behavior was observed in both sputum and blood samples, that it is a consequence of normal sex-differences. On the other hand, there is a relatively smaller subset of transcription factors – those with negative correlation coefficients – whose sexually-dimorphic targeting of these important functions is *opposite* in the sputum and blood networks. We indicate the 23 transcription factors with correlation less than −0.4 by arrows in Figure 6A.

The transcription factors most differentially-targeting these key functions between sputum and blood, based on our correlation measure, include the HAND1::TCFE2A complex, FOXF2, PAX4, the MYC::MAX complex, and SOX5 (Figures 6C-G). Both FOXF2 and SOX5 have been implicated in COPD or lung biology and it is interesting that we observe them in this sex-specific context. For example, FOXF2 has been shown to quantitatively increase binding upon smoke exposure in female mice [58] and modulates the expression of lung genes [59]. SOX5 is a candidate for COPD susceptibility and important for lung development [60].

### A network model for sex-specific targeting of functionally-related genes in COPD

The GSEA analysis we have performed based on the differential-targeting of genes is clearly very powerful and has led to the identification both of potential biological functions targeted in a sexually-dimorphic manner in COPD as well as several transcriptional regulators that may be mediating those differences. One strength of this analysis is that it relies upon characterizing network differences based on relative changes in targeting patterns. However, in doing so it also ignores the actual strength of predicted network interactions. In other words, if a gene is more targeted in one ensemble of networks relative to the other, that gene is highly implicated in the GSEA analysis, even if its input edges have low absolute edge-weight values predicted across all the networks in both ensembles. It is unlikely that the systemic differential-targeting of functions we see across our panel of transcription factors in Figure 6A actually corresponds to multiple strong regulatory interactions from every one of them.

**Figure 7:**
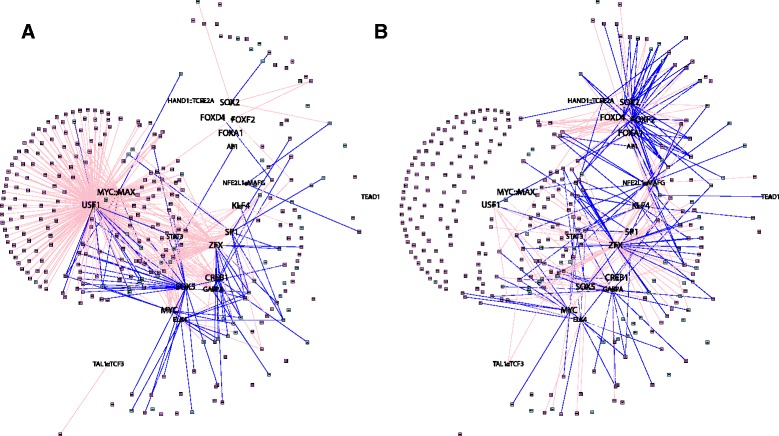
**Illustrations of core subnetworks of sex-specific regulation in COPD. (A)** The sputum subnetwork, defined by sputum sex-specific edges (as shown in Figure 2B) that extend between the identified disease-specific drivers (Figure 6B) and genes annotated to one of the top five differentially-targeted GO categories in ensembles of networks built based on sputum expression data (Figures 4B and 6). **(B)** The blood subnetwork, defined by blood sex-specific edges (Figure 2C) that extend between the identified disease-specific drivers (Figure 6B) and genes annotated to top five GO categories identified as differentially-targeted between male and female networks (Figures 4B and 6). Edges and genes are colored pink or blue based on whether they were identified as part of the female or male networks or functions, respectively.

To better appreciate the relationship between likely regulatory interactions and the results of our functional analysis, we next visualized subnetworks based on the female-specific and male-specific edges we previously identified (Figures 2B-C). In order to interpret our functional results in this regulatory network context we identified sex-specific edges that extend between the 23 disease-specific transcription factors and genes annotated to the top differentially-targeted functions. We illustrate the resulting subnetworks in Figure 7. Edges and genes are colored pink or blue based on whether they were identified as part of the female or male networks or functions, respectively.

We observe that some transcription factors, such as CREB1 and ZFX target distinct sets of genes in both the male and female sputum networks. ZFX is the X-linked version of a protein that plays a role in molecular sex determination [61], so it may not be surprising that we found sex-specific differences in its regulatory patterns. However, it has also been implicated in lung cancer [62,63]. Similarly CREB is over-expressed in many cancers, including lung cancer [64], and, interestingly, has been shown to interact with the estrogen receptor and to have age and sex- dependent expression patterns in the human brain [65].

Several transcription factors dominate in one sex compared to the other. For example, the MYC::MAX complex appears to primarily target genes annotated to functions enriched in the female-specific sputum network (but not in the blood network) while SOX5 targets genes in the male-specific sputum network. USF1, in particular, appears to be a “hub” transcription factor for the female functionally related-genes in the sputum networks.

USF1 both regulates and interacts directly with estrogen receptor (ER) in a protein complex [66], which may explain its female-specific activity. Estrogen has also been shown to induce USF1 to bind to the regulatory regions of several genes [67-69]. USF1 is involved in the cross-talk between hypoxia-related elements such as Aryl hydrocarbon receptor (AHR) and the estrogen receptor, inhibiting the former [70-72]. This relationship may be important in sex-specific COPD biology as AHR has previously been identified as potentially important for sex-specific differences in lung cancer [73].

Sex-specific effects of USF1 have been noted previously [74,75]. Consistent with our findings, it has been reported that in mouse liver, male gene signatures are enriched for functions such as immune response while female signatures are enriched in functions such as oxidoreductase activity and mitochondrion [75]. ChIP of USF1 in HepG2 cells also indicates that it regulates nuclear mitochondrial genes [76]. Most interestingly, however, is that fact that USF1 has been shown to bind to the promoter and mediate the expression of PGC-1α [77,78] which, as we previously noted, is an important regulator of mitochondrial biogenesis [57], and, along with several PPARs, has been shown to be expressed at lower levels in the skeletal muscle of COPD patients [55]. Therefore, it is not unreasonable to suppose that USF1, as indicated by our PANDA network analysis, may be an important mediator of mitochondrial-activity in a sexually-dimorphic manner in patients with COPD.

## Conclusions

In this study we identified functionally related sets of genes that are strongly differentially-targeted between men and women with COPD. Our results suggest that sexual dimorphism in features of COPD may be a consequence of the re-wiring of cellular networks around particular biological pathways, especially those involved in mitochondrial function and energy metabolism, leading to differences in COPD in men and women. Although these functions have previously been implicated in COPD, little is known about their disease- and sex-specific regulation. In addition, despite the fact that there is a large body of research concerning the structural features of *individual* regulatory networks [79-82], quantifying *differences* in network features is relatively understudied and there are few systematic approaches for characterizing variability in gene targeting. In our analysis we contrasted networks and identified functionally related sets of genes that are strongly *differentially-targeted* between men and women with COPD.

One of our most striking findings was clear differential-targeting patterns in the absence of similarly compelling differential-expression. Several potential biological mechanisms may play a role in mediating this differential targeting. One possibility is that multiple transcription factors compete for the same binding site upstream of a given target gene, but which one primarily regulates that gene is dependent on the cellular context (for example a change in protein abundance or conformation in response to sex hormones). Another possibility is that several transcription factors have potential binding sites upstream of a gene, but in females certain sites are inactive (for example through an epigenetic factor or a mutation) and in males others are inactive.

Using a network-based approach we were able to identify potential sex- and disease-specific transcriptional regulators of these biological functions, the most striking of which was USF1. Although USF1 has previously been implicated both in the regulation of nuclear mitochondrial genes and in sexual-dimorphism, its specific role in COPD is largely unknown and our findings are an important step in beginning to understand its potential importance. Curiously, an increase or decrease in *overall* out-degree by transcription factors between male and female networks did not always directly correspond to differential-targeting of particular biological *functions* between male and female networks. For example USF1 had an overall higher out-degree in male networks (Figure 3E), yet it also had increased targeting of mitochondrial functions in female networks (Figures 6 and 7). This highlights the importance of interpreting network measures within a functional context.

As with any computational analysis, there are limitations in our investigation that result from the underlying data we used; for example the number of genes included on the expression array may affect the comprehensiveness of the information incorporated in the model. One limitation in our specific application is that, although we found many sex-specific regulatory features, the sputum and blood expression data we used was only collected from individuals with COPD, and thus we lacked truly “normal” controls—this is a crucial direction for future research. However, by focusing on sex differences we observed just in the sputum networks and not the blood networks, we believe our findings are likely to represent sex-specific network alterations that are important for COPD. We also used a covariate-free model to evaluate differential-expression in order to be consistent with our subsequent regulatory network analysis, which does not directly model the role of covariates. It is therefore possible that in addition to the sex-specific regulatory changes we observe, there may also be gene expression differences between men and women with COPD that are simply not captured using a covariate-free approach. However, we suggest that is equally likely that similar outcomes in gene expression are mediated by distinct sets of transcriptional regulators. For example, it is reasonable to imagine that sex hormones (such as estrogen), which we only modeled in our network through receptor binding sites, might change the functions of some transcription factors (for example USF1) in other ways, requiring cells to respond and differentially rewire the effected portion of their regulatory network in order to maintain viability. In this case the overall expression profile of the cells might be similar, but the factors mediating that response could be vastly different.

Genomic assays, such as gene expression data, provide a snapshot of the state of a cell and most widely used analysis approaches identify differences in *individual genes* by collectively comparing groups of samples. We believe one limitation of gene-centered approaches, especially in the context explored here, is due to the fact that individual genes do not define the biological processes that drive cell states, but that phenotypic alterations are better characterized by networks of interactions linking genes. In contrast, our *network approach*, although complementary to differential gene expression analysis, highlights fundamentally different aspects of sex-specific biology. Namely, that a gene, or a collection of genes involved in a biological function, may be similarly *expressed* in both men and women, but this expression may be regulated by different upstream factors. Understanding how the *targeting* of biological functions is distinct between sexes in COPD helped to elucidate potentially sexually-dimorphic mechanisms of the disease, an endeavor with relevance for both sex-specific diagnostics and therapeutics. Differential targeting of biological pathways is likely not limited to sex-specific disease features, and we believe the methods we employ here will be widely applicable to better understanding other biological systems and diseases.

## Methods

### Building ensembles of PANDA networks using a jack-knife

We used PANDA [21] to integrate expression information with transcription factor motif and protein-interaction data. In our analysis we parsed the expression data by sex, employed a jack-knifing procedure and ran PANDA multiple times to construct sets of sex-specific, genome-wide transcriptional regulatory networks. The specifics of how we processed the input data and reconstructed the PANDA network models are included below:

#### Expression data

We obtained the CEL data files for 264 expression experiments performed on blood and sputum samples collected from 132 individuals and profiled using Affymetrix HGU 133 plus2 microarrays. We RMA-normalized the expression data in R [26,83], and mapped probes to Entrez-gene IDs using a custom CDF [27]. These data include 18960 probes sets, mapping to 18895 unique genes (based on Hugo Gene Symbols). 15820 of these genes are also included in our motif scan (see below), including 651 on the sex chromosomes. After an initial PCA analysis investigating the clustering of samples based on the expression of genes on the Y chromosome, we excluded genes on the sex chromosomes and removed expression samples for 6 individuals who did not cluster correctly according to sex. We used expression data for the remaining 126 individuals (42 females and 84 males) and 15169 genes when constructing sex- and tissue-specific genome-wide regulatory networks. We also created a “random” version of the sputum expression data by permuting autosomal gene labels.

#### Motif data

We obtained position weight matrixes (PWM) for 130 core vertebrate transcription factor motifs from JASPAR [84,85]. To identify the target locations for each motif, each candidate sequence S was given a motif score equal to log [P(S|M)/P(S|B)], where P(S|M) is the probability of observing sequence S given motif M, and P(S|B) is the probability of observing sequence S given the genome background B. We modeled the distribution of these motif scores by randomly sampling the genome 10^6^ times. Motif sites with a significance level of p < 10^−5^ and that fell within the promoter region ([−750, 250] base-pairs around the transcriptional start site) of one of the genes measured on our expression arrays were used to defined as an edge between a motif and that gene in our regulatory network prior.

It is important to note that although when building our primary network models we did not include genes on the sex chromosome as potential targets, we did *not* remove motif information for sites bound by transcription factors encoded on the sex chromosomes (such as AR on chrX and SRY on chrY). We reasoned that since the motif sequences for these transcription factors still exist in the regulatory regions of autosomal genes they can still be indicative of information about a target gene’s local regulatory network structure.

#### PPI data

Interactions between human transcription factors were obtained from the supplemental material of [34]. We excluded interactions in this set when either transcription factor in the interaction did not directly match one of the motifs included in our regulatory network prior.

#### Reconstructing PANDA networks

To construct multiple sex- and tissue- specific network models, we selected ten subjects of the same sex at random, identified the sputum, blood and “random” expression data associated with these subjects, and used PANDA to, separately, integrate each of these three sample-sets of expression data with motif and protein-protein interaction data. We did multiple random selections of subjects of the same sex, constructing one hundred female-specific and one hundred male-specific networks for each tissue.

### Identifying differentially-called edges between network ensembles

PANDA reports the probability that an edge exists between a transcription factor (i) and gene (j) in an estimated network (n) as a Z-score (*Z*_*ij*_^*(n)*^). To select edges that are differentially-called between male and female network ensembles, for each edge we calculated (1) its average edge-score across all networks in each of the two ensembles, (2) the difference between these average scores, and (3) used a t-test to evaluate the significance of the difference in edge-score distribution between the male and female network ensembles. We corrected this significance for multiple hypothesis testing. To select edges distinct between female and male network ensembles, we then selected edges that had an average edge score greater than zero in at least one of the ensembles, an absolute edge score difference of at least 0.25, and an FDR significance less than 10^−5^.

### Clustering network differences

In order to better appreciate large scale patterns in male/female sputum regulatory network differences, we performed a hierarchical clustering on a transcription factor by gene matrix populated with the t-statistic value of the corresponding network edge, calculated when comparing the distribution of predicted edge scores across the male versus female sputum network ensembles. Hierarchical clustering was done separately in each dimension using one minus the Pearson correlation as the distance metric and the “complete” linkage method.

### GSEA

To run GSEA in a consistent manner on both gene expression and network regulatory data, we downloaded the java command line version of the program from www.broadinstitute.org/gsea/ . We ran GSEA permuting gene set labels. Further, in order to ensure consistency between the GSEA analysis and the functional enrichment analysis we performed using Fisher’s exact test (shown in Figure 2H-J), we ran the GSEA program using custom Gene Matrix Transposed (GMT) files that we constructed from human GO annotations downloaded from geneontology.org (access date: 02/02/13).

In analyzing the GSEA results, we consider the FDR p-values reported by GSEA. Specifically, we report enrichment in female categories based on the –log_10_(FDR) significance (resulting in positive values), and enrichment in male categories based on the + log_10_(FDR) significance (resulting in negative values).

## Additional files

Additional file 1:
**Supplemental text and figures.** This file includes PCA plots, the results of a gene expression and network-based analysis performed without removing sex chromosome genes and the results of several additional network analyses that complement those described in the main text. Additional file 2:
**List of genes that have significantly different variance in their sputum expression profiles when comparing females and males.**
