# Supplementary material for: Sexually-dimorphic targeting of functionally-related genes in COPD
Source: BMC Syst Biol. 2014 Nov 28;8:118. doi: 10.1186/s12918-014-0118-y (PMC4269917; doi:10.1186/s12918-014-0118-y)
Supplement: Additional file 1: — Supplemental text and figures. This file includes PCA plots, the results of a gene expression and network-based analysis performed without removing sex chromosome genes and the results of several additional network analyses that complement those described in the main text. [file 12918_2014_118_MOESM1_ESM.pdf]

Supplemental Material For: Sex-specific Differential-Targeting of Functionally-Related Genes in COPD

Analysis without Removing Sex Chromosome Genes

Differential Expression Analysis

We tested to see what genes were strongly differentially-expressed between males and females in either sputum or blood samples, using an unpaired two-sample t-test. This analysis differs from that in the main text in that it includes *all genes* instead of only autosomal genes. In this analysis we find a number of genes that are significantly (FDR<0.1) differentially-expressed between males in females in sputum (20 higher in males and 30 in females) and in blood (44 higher in males and 39 in females). These genes are listed in Supplemental Tables 1 and 2.

The majority of differentially-expressed genes found in this analysis are sex-chromosome genes. We note that because the false discovery rate is applied to a set of p-values, rather than an individual p-value, the values calculated for an individual comparison depend upon the other p-values included in the rest of the set. Consequently, the values listed in these tables differ slightly from the values we found when performing an FDR correction when only including autosomal genes (see Figure 1C in the main text) and explains why some autosomal genes appear in the results of this analysis, but not in the autosomal-only analysis included in the male text.

This serves as a positive control on the quality of the expression data since the results are consistent with what we might expect when comparing males and females, with many chrY genes more highly expressed in males, and many chrX genes more highly expressed in females (with the exception of NDP in the sputum comparison and KAL1 in the blood comparison).

Network Analysis

We also ran our jack-knifing network reconstruction and subsequent comparisons without removing genes on the sex chromosomes. In this analysis we focused only on the sputum expression data. We constructed 100 male and 100 female networks, using the same random selections of patients as in the main text. We then calculated the in-degree of all genes in these network ensembles and identified the top most differentially-targeted genes in these networks (Supplemental Figure 2A). Overall, the differentially-targeted genes identified in this analysis include the ones identified in the main text analysis as well as several chrX genes more highly targeted in the female network ensembles and chrY genes more highly targeted in the male network ensembles. When including allosomal genes in the network reconstruction, the two genes with the highest increased targeting in the female compared to the male networks are XIST and TSIX. Both of these genes are located on the X chromosome and are involved in sex-specific X-inactivation. The two genes with the highest increased targeting in male compared to female networks in this analysis, NCRNA00185 and USP9Y, are located on the Y chromosome.

We also investigated the differential-targeting patterns around all genes included in this network reconstruction that are located on the Y chromosome (Supplemental Figure 2B). Although not all chrY genes are identified as *strongly* differentially-targeted between male and female network ensembles, all but three are identified as more highly targeted in the male networks compared to the female networks. This “false positive” rate of differential-targeting is

commensurate with the results of differential-expression analysis, as four chromosome Y genes are identified as more highly expressed in females compared to males. This includes RBMY2FP, XGPY2, PCDH11Y and TBL1Y. Although none of these genes are located in the pseudoautosomal regions of the sex chromosomes, XGPY2, PCDH11Y and TBL1Y all have homologues on the X chromosome [1, 2].

It is interesting to note that the chromosome Y genes that have relatively weaker differential-targeting patterns between male and female networks are also not significantly differentially-expressed between males and females. Thus for genes on the sex chromosomes, differential-targeting is highly concordant with differential-expression, as one might expect.

Differential-targeting Analysis Permuting Sample Labels

We also performed an analysis to verify that the differential targeting we observe in the main text is significant with respect to permuting sample labels. To begin, we constructed a single female PANDA network using all 42 female sputum gene expression samples, and a single male PANDA network using all 84 male sputum gene expression samples. We then estimated 200 additional networks by randomly permuting the sex-labels of the samples one hundred times and reconstructing networks for splits of the data into “42 random” and “84 random” gene expression samples.

For each of these 202 networks (the male and female networks plus the 100 pairs of sex-label permuted networks) we calculated the degree of each gene (sum of edges pointing to that gene). To obtain an estimate of the differential-targeting of genes between the female and male networks, we calculated the “differential-degree” of each gene by subtracting the degrees of the genes in the male network from the degrees of the genes in the female network. We also calculated the differential-degree for the “42 random” versus “84 random network” pairs. For the 25 most-differentially targeted genes identified in Figure 3 of the main text we report the differential-degree in the female versus male network comparison (Supplemental Figure 4A). We see that these genes have strong differential-degree between the male and female network and that this is much greater than we might expect upon a permutation of the sex-labels.

Next we used the gene differential-degree values calculated for each of these 101 network pairs to perform 101 pre-ranked GSEA analyses. We then selected the top ten categories identified in the female/male network comparison and show the normalized enrichment scores (NES) calculated by GSEA for these functions (Supplemental 4A). Those that are also considered significantly enriched (FDR<0.01) are noted with an asterisk. The functional categories identified in this analysis are highly similar to those presented in the main text. We also calculated and show the mean and standard deviation of the NES across the one-hundred permuted network comparisons. We see no systematic enrichment for functional categories across the sex-permuted networks. It is also reassuring to note that the categories identified as significant based on the FDR (the ones with asterisks) have the strongest differences between the NES calculated in the male/female network GSEA analysis when compared to the distributions of the NES values calculated across the 100 sex-permuted GSEA analyses.

Covariate-Matched Network Ensemble Analysis

We also implemented an approach to match covariates between sets of jack-knifed gene expression samples. Our approach followed these seven steps:

1) Pick a sample at random. Determine if it is male or female.
2) Identify all samples of the opposite sex with the same GOLD stage.

3) From those samples, identify the sample with the most similar (age+pack-years).
4) Repeat 1-3 ten times, resulting in ten “paired” samples.
5) Compare, statistically by unpaired t-test, both the age and the pack-years in these ten male and ten female samples.
6) If both age and pack-years are NOT statistically different (p-value>0.2), keep the paired set, otherwise, repeat 1-5.
7) Repeat 1-6 100 times.

The result of this was 100 paired sets of gene expression samples, where each paired set consisted of ten male samples and ten female samples with identical GOLD stage make-up and no significant differences in age or pack-years. One limitation of this approach is that this stringent matching results in a strong bias for re-sampling some of the subjects many times and others much fewer.

We repeated the ensemble network analysis in the sputum gene expression dataset, building PANDA networks for each of these covariate-matched sample sets. We ran GSEA on the degree of the genes in these covariate-matched networks just as we did in the main text and observe that the most differentially-targeted functional categories are nearly identical to before. The calculated FDR values, although still very significant, are slightly less so compared to the non-matched analysis presented in the main text (Supplemental Figure 6).

Jack-knifed differential-expression compared to jack-knifed differential-targeting

The jack-knifing approach we used to compare network features differs from a standard differential-expression analysis in several ways. First, when evaluating the differential-expression we included 42 female and 84 male samples; however, in order to help alleviate the differences in sample sizes between the sexes when we evaluated differential-targeting we included 100 female and 100 male networks (so more networks compared to expression samples). More significantly the differential-expression and differential-targeting analyses also differ in that the gene expression samples represent 126 independent measurements, whereas, due to the jack-knifing procedure we used to reconstruct our networks, the 100 female and 100 male networks are not truly independent.

To more similarly compare differential-expression and differential-targeting using GSEA, we have additionally run GSEA one hundred times to quantify the differential-expression of functionally-related groups of genes within the same sets of 10 female and 10 male expression samples that we used to reconstruct each of our jack-knifed gene regulatory networks. We report the top twenty male and female categories identified in this analysis based on the average NES across the 100 GSEA runs in Supplemental Figure 7A.

To evaluate how these results compare to performing 100 differential-targeting analyses on networks reconstructed from these sets of 10 female and 10 male samples, we also ran one hundred pre-ranked GSEA analyses based on the differential-degree of the genes between the female and male networks reconstructed from each of these 100 expression sample sets (for a definition of differential-degree see “Differential-targeting Analysis Permuting Sample Labels” above). The top categories based on the average NES are shown in Supplemental Figure 7B. These categories are highly consistent with what we observed in the analysis presented in the main text (see Figure 4). Comparing between Supplemental Figure 7A and 7B we also can see that the networks again appear to have very strong differential-targeting patterns while the differential-expression is fairly non-compelling.

Supplemental Figure Legends

Supplemental Figure 1 – (A) A plot of the first two principle components resulting from a principle component analysis on the chrY gene expression across 264 blood and sputum samples collected from 132 individuals with COPD. Six samples were identified as not clustering correctly based on sex (circled) and the data associated with these subjects was removed in the analysis performed in the main text. (B) A plot of the first two principle components resulting from a principle component analysis on all autosomal gene expression across 126 sputum samples. Samples are colored based on the subject sex, age, pack-years or COPD GOLD stage.

Supplemental Figure 2 – Summary of network analysis results when network ensembles are reconstructed using expression data that includes genes on the sex chromosomes. (A) The top most differentially-targeted genes (analogous figure to Figure 3C in the main text). Genes located on the X and Y chromosomes are bolded and colored pink and blue, respectively. (B) The differential-targeting patterns around genes on the Y chromosome. X/Y designates the pseuodautosomal region. The significance of differential-expression and differential-targeting was calculated using an unpaired two-sample t-statistic, with positive t-statistic values indicating increased expression or targeting in females compared to males and negative t-statistic values indicating increased expression in males compared to females.

Supplemental Figure 3 – An illustration of how similarity in expression levels between two sets of samples, for example males and females, can result in different co-expression patterns. PANDA uses co-expression as an initial estimate for gene co-regulation in order to identify potential transcription factor drivers of these patterns when building gene regulatory networks.

Supplemental Figure 4 – Analysis of network properties when comparing PANDA-reconstructed male and female regulatory networks and when comparing regulatory networks reconstructed after permuting the sex-labels of the samples. (A) The differential-degree of genes in the female versus male networks (values indicated) as well as the differential-degree of genes across one hundred sample-permuted networks (mean plus or minus the standard deviation indicated). (B) The normalized enrichment score for the top ten male and female categories identified as differentially-targeted between the female and male network according to a pre-ranked GSEA on differential-degree. An asterisk in the first column indicates that the category was found as significantly differentially-targeted (FDR<0.01) in the male/female network comparison.

Supplemental Figure 5 – For each subject we averaged the expression levels of the genes annotated to the identified highly differentially-targeted functional categories (see Figure 6 in the main text). In this figure we present box plots showing the distribution of those values in female and male subjects at different COPD stages. We see a strong association between the expression of these genes and COPD stage, especially for the genes annotated to the categories more highly targeted in female compared to male networks.

Supplemental Figure 6 – The FDR significance of top ten female and male differentially-targeted functional categories from Figure 4B of the main text, as reported by GSEA when evaluating the networks reconstructed from the random sampling approach used in the primary analysis presented in the main text (first column), as well as for networks reconstructed using a covariate-matched sampling approach (second column).

Supplemental Figure 7 – The normalized enrichment scores from GSEA analyses run on (A) 100 sets of expression samples, each consisting of 10 females and 10 males, (B) the differential-degrees of genes in 100 pairs of networks, reconstructed from the same set of 10 female and 10 male samples. In both cases the twenty top male and female categories (based on the average NES across the GSEA runs) are shown.

Supplemental Table Legends

Supplemental Table 1 – List of the significantly differentially-expressed genes (FDR<0.1) in sputum when we include genes located on the sex chromosomes in the analysis.

Supplemental Table 2 – List of the significantly differentially-expressed genes (FDR<0.1) in blood when we include genes located on the sex chromosomes in the analysis.

References

1. Weller PA, Critcher R, Goodfellow PN, German J, Ellis NA: **The human Y chromosome homologue of XG: transcription of a naturally truncated gene.** *Hum Mol Genet* 1995, **4:**859-868.
2. Wilson ND, Ross LJ, Close J, Mott R, Crow TJ, Volpi EV: **Replication profile of PCDH11X and PCDH11Y, a gene pair located in the non-pseudoautosomal homologous region Xq21.3/Yp11.2.** *Chromosome Res* 2007, **15:**485-498.

Supplemental Figure 1

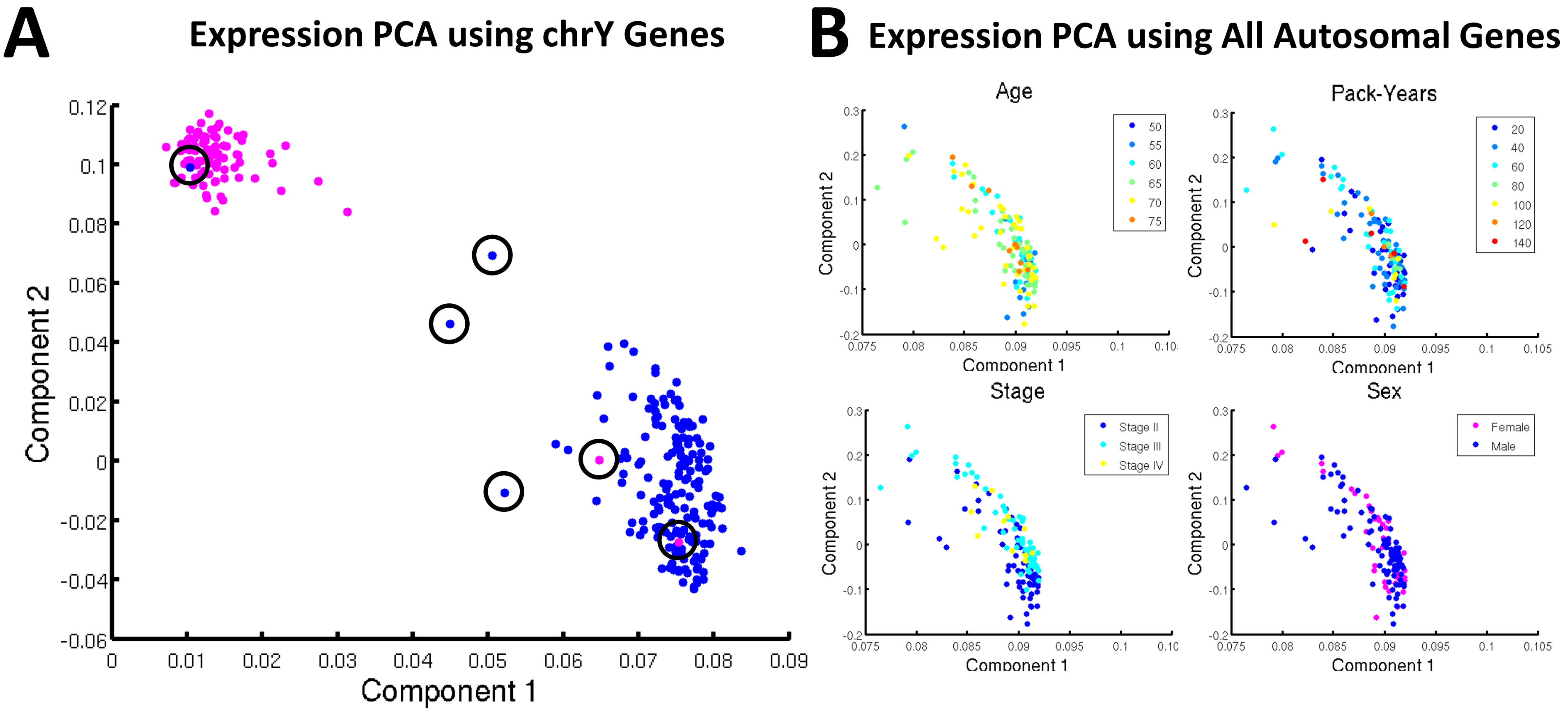

Supplemental Figure 2

A

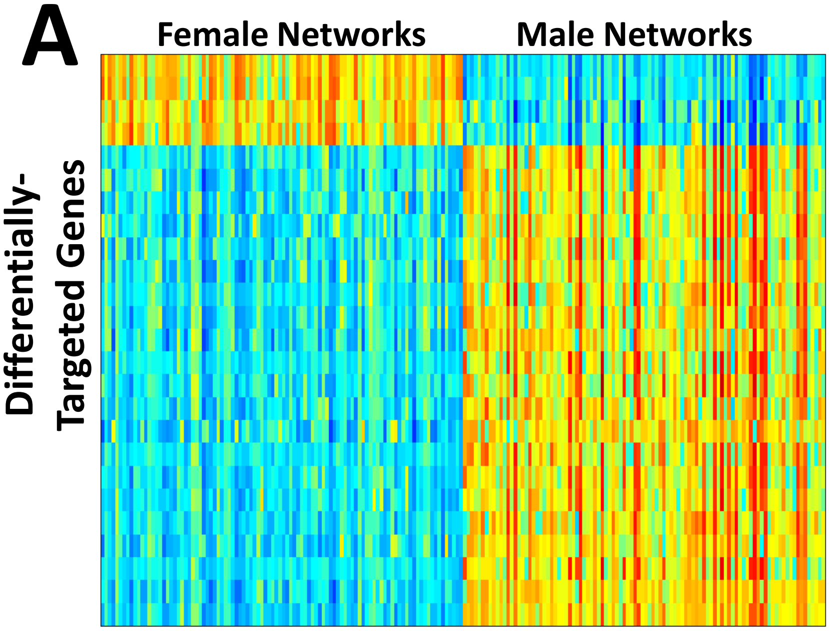

Gene Name	Diff-k FDR	Diff-k T-stat	Diff-Exp FDR	Diff-Exp T-stat
XIST	1.61E-61	25.53848	2.08E-104	79.56387
TSIX	7.42E-47	20.06777	2.11E-92	62.83543
WDR85	1.42E-38	17.14311	0.93619	-1.03589
ZNF628	1.22E-35	16.12607	0.869626	-1.44119
PIGB	4.30E-33	-15.2443	0.194007	3.363222
SPAST	3.35E-33	-15.2854	0.54572	2.305187
ZNF700	2.38E-33	-15.3398	0.491676	2.432248
JAK2	2.21E-33	-15.3569	0.718683	1.865552
PPP2R2A	5.68E-34	-15.5565	0.954967	0.229607
KIAA1468	4.96E-34	-15.583	0.54472	2.312821
DGCR11	2.48E-35	-16.0171	0.633151	2.091103
ERP27	3.27E-36	-16.3228	0.390427	2.665265
C1GALT1	3.22E-37	-16.664	0.33186	2.848021
ZDHHC17	5.13E-38	-16.9375	0.65812	2.027297
TNFSF13B	1.45E-38	-17.1296	0.553492	2.281847
PDS5B	7.37E-43	-18.5933	0.599101	2.171757
RPS4Y1	5.69E-44	-18.9846	2.14E-103	-77.7926
LPCAT2	3.26E-44	-19.0814	0.869774	1.441305
ZCCHC7	1.43E-44	-19.2192	0.662122	2.004938
HSDL2	1.33E-44	-19.2471	0.08125	3.806909
PRKY	4.40E-46	-19.7767	2.86E-27	-15.315
TTTY15	3.87E-47	-20.1936	8.89E-46	-24.2839
HNRNPU	4.77E-47	-20.1957	0.857063	1.486581
USP9Y	1.38E-64	-26.809	3.22E-56	-30.5126
NCRNA00185	3.18E-67	-27.9912	3.72E-30	-16.5981

B

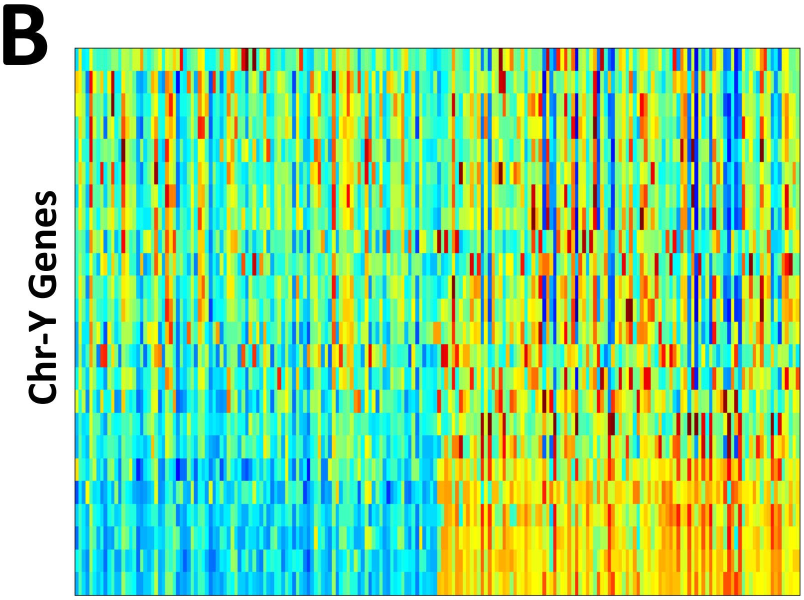

Gene Name	Diff-k FDR	Diff-k T-stat	Diff-Exp FDR	Diff-Exp T-stat
RBMY2FP	0.122945	0.658727	0.954946	0.249118
TTTY11	0.189582	0.172642	0.883512	-1.37153
RBMY3AP	0.206783	0.049388	0.923812	-1.16933
TTTY14	0.188921	-0.17819	0.951936	-0.33699
XGPY2	0.170158	-0.3134	0.875361	1.410231
AMELY	0.168911	-0.32202	0.953676	-0.45148
SRY	0.120144	-0.6797	0.952837	-0.49663
TTTY5	0.080473	-1.0182	0.953024	-0.37344
PCDH11Y	0.035009	-1.56991	0.877879	1.404743
NLGN4Y	0.019338	-1.89083	0.521695	-2.33997
TTTY12	0.012775	-2.09258	0.883522	-1.36965
TBL1Y	0.004638	-2.5314	0.950145	0.744002
TTTY10	0.000821	-3.16449	6.77E-16	-10.55
EIF1AY	0.000812	-3.16837	6.44E-105	-80.7943
TMSB4Y	6.93E-07	-5.07229	0.001748	-4.95582
ZFY	6.03E-12	-7.44384	6.88E-53	-28.4019
DDX3Y	1.79E-14	-8.48849	2.45E-67	-38.3828
UTY	7.42E-15	-8.64051	3.31E-69	-39.9035
KDM5D	8.04E-33	-15.1393	1.83E-95	-66.7454
RPS4Y1	5.69E-44	-18.9846	2.14E-103	-77.7926
PRKY	4.40E-46	-19.7767	2.86E-27	-15.315
TTTY15	3.87E-47	-20.1936	8.89E-46	-24.2839
USP9Y	1.38E-64	-26.809	3.22E-56	-30.5126
NCRNA00185	3.18E-67	-27.9912	3.72E-30	-16.5981

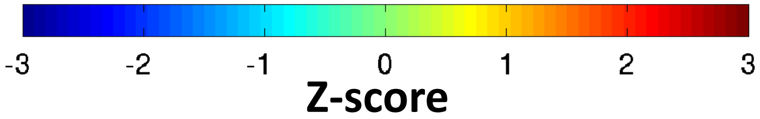

Supplemental Figure 3

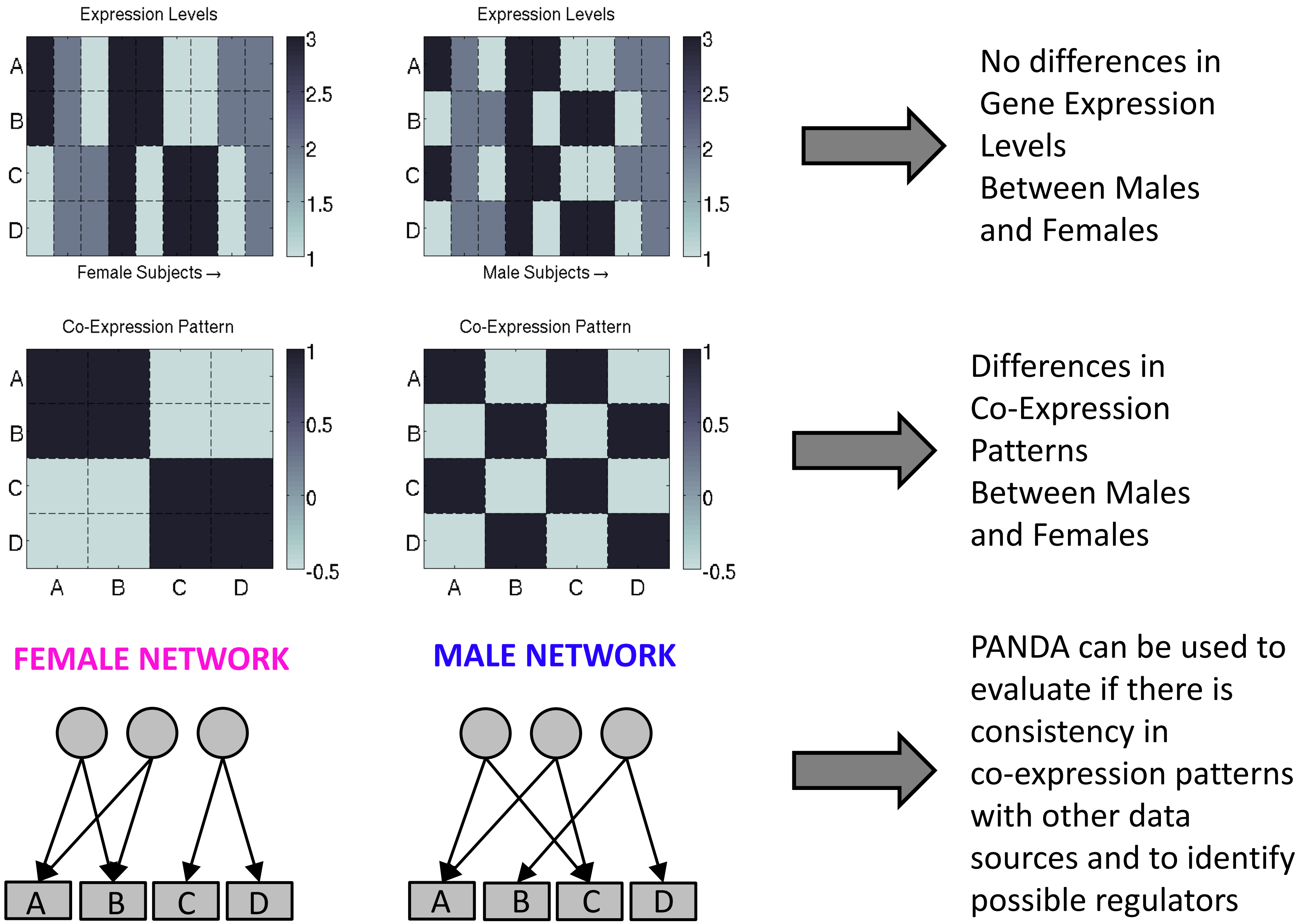

Supplemental Figure 4

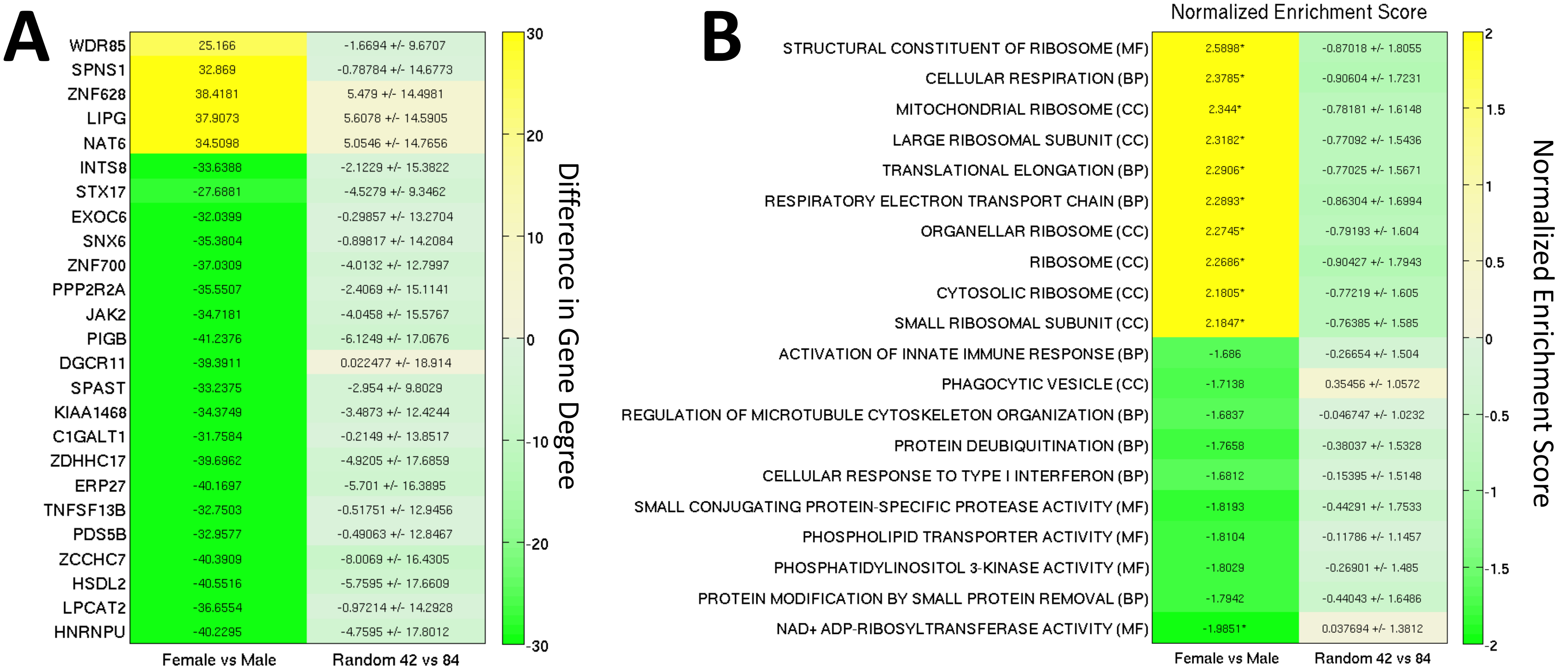

Supplemental Figure 5

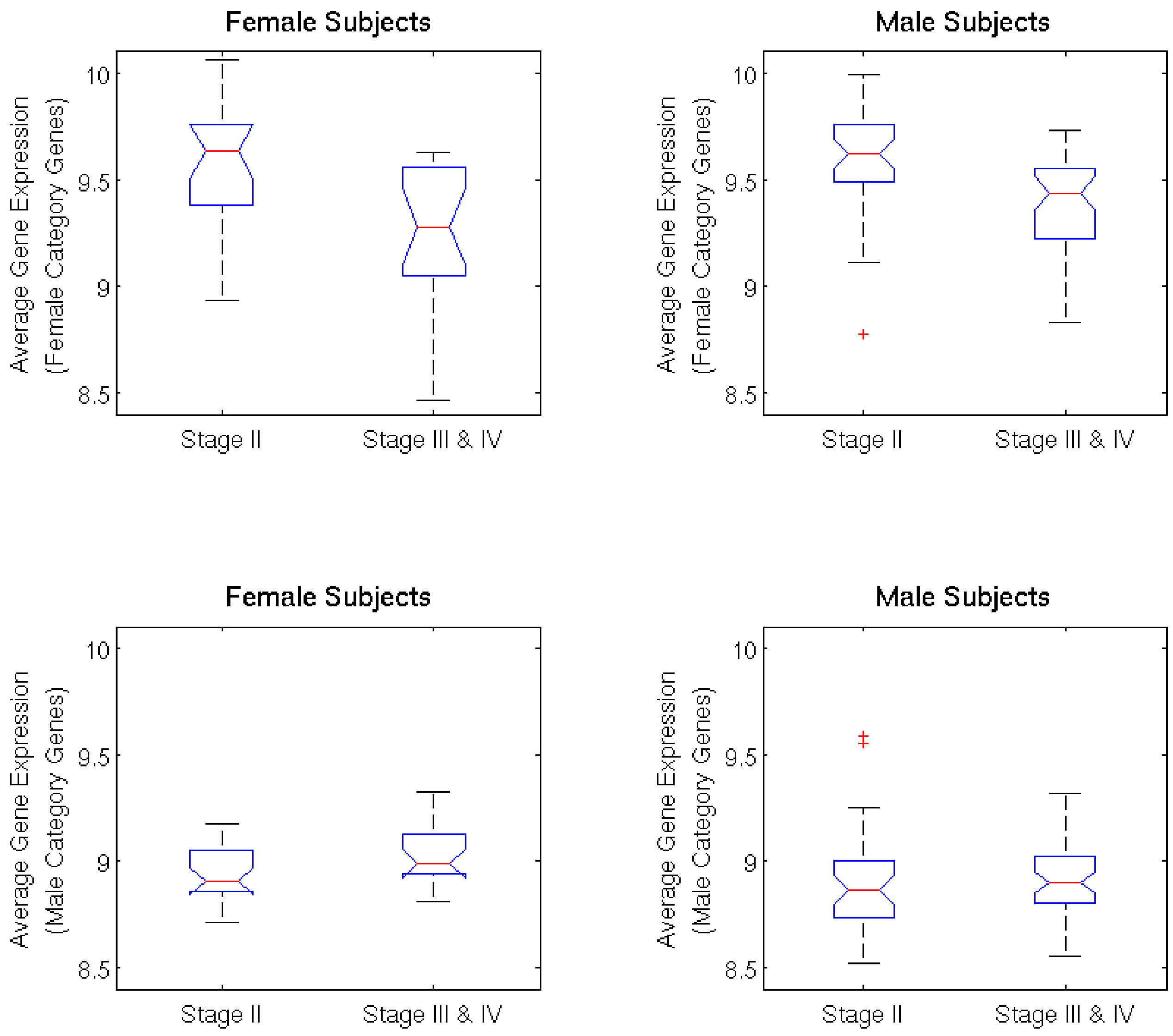

Supplemental Figure 6

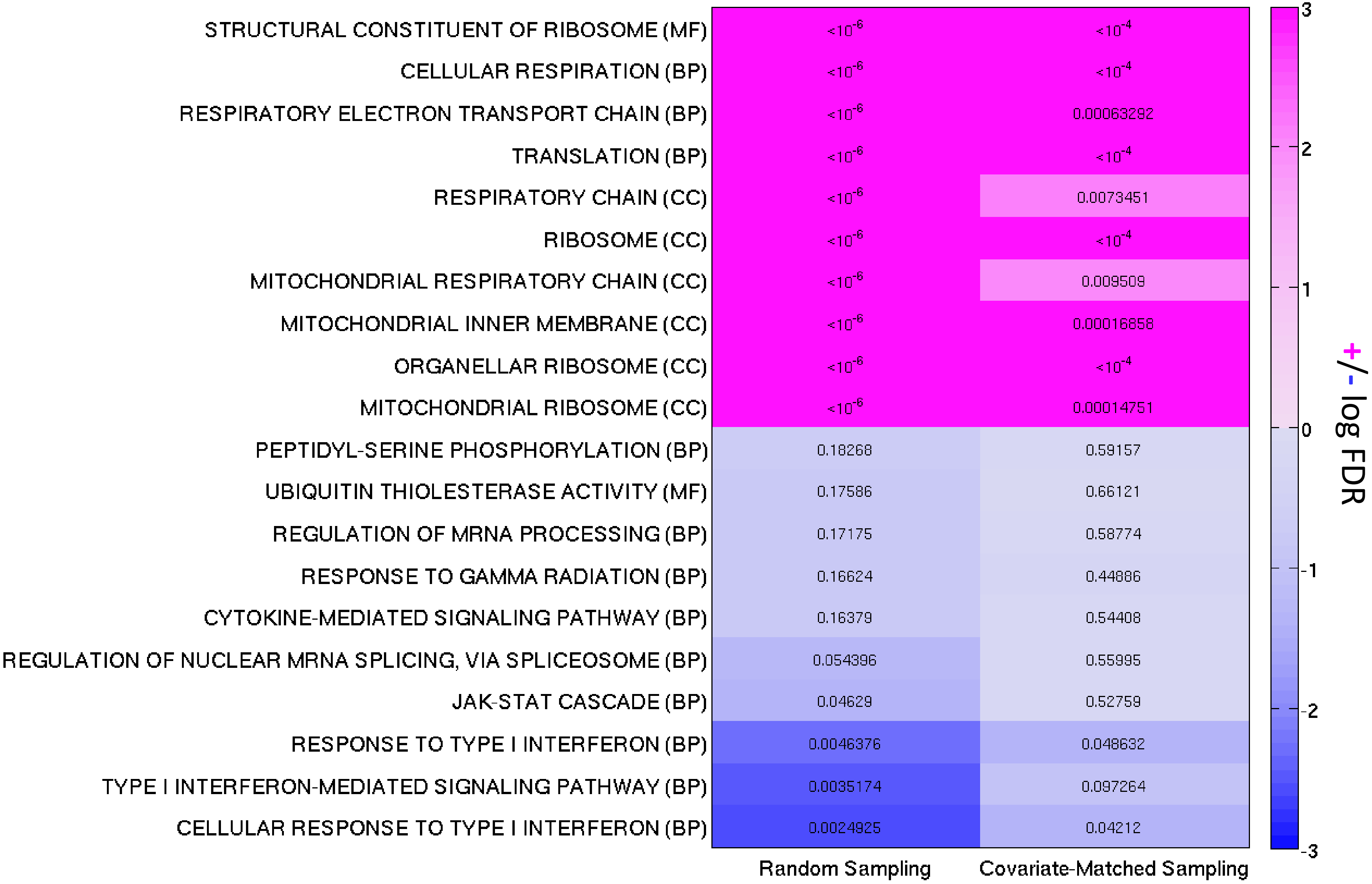

Supplemental Figure 7

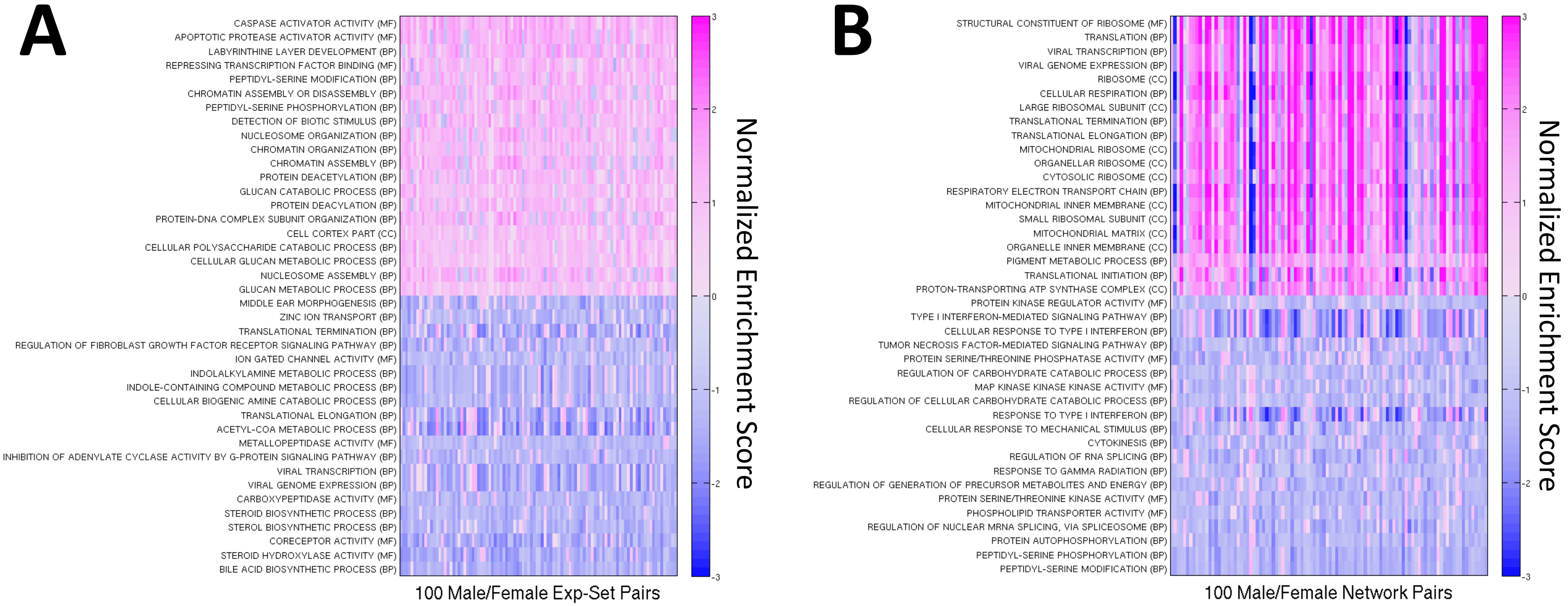

Supplemental Table 1

Differentially-Expressed Genes in Sputum Samples, including genes on the Sex Chromosomes

More Highly Expressed in Males

Gene	pvalue	FDR	FC	chr
EIF1AY	4.25E-109	6.44E-105	0.34	Y
RPS4Y1	4.25E-107	2.14E-103	0.40	Y
KDM5D	4.84E-99	1.83E-95	0.37	Y
UTY	1.31E-72	3.31E-69	0.60	Y
DDX3Y	1.13E-70	2.45E-67	0.53	Y
USP9Y	1.70E-59	3.22E-56	0.50	Y
ZFY	4.09E-56	6.88E-53	0.50	Y
TTTY15	5.87E-49	8.89E-46	0.64	Y
NCRNA00185	2.70E-33	3.72E-30	0.71	Y
PRKY	2.26E-30	2.86E-27	0.84	Y
TTTY10	5.81E-19	6.77E-16	0.82	Y
TMSB4Y	2.31E-06	1.75E-03	0.97	Y
WSB2	5.66E-05	3.57E-02	0.98	12
S1PR3	1.52E-04	6.96E-02	0.93	9
STARD3NL	1.49E-04	6.96E-02	0.98	7
SPRED2	1.80E-04	7.51E-02	0.97	2
SNX9	1.83E-04	7.51E-02	0.97	6
NDP	1.89E-04	7.52E-02	0.87	X
NRAS	2.21E-04	7.97E-02	0.97	1
KCNN4	2.44E-04	8.60E-02	0.95	19

More Highly Expressed in Females

Gene	pvalue	FDR	FC	chr
XIST	2.75E-108	2.08E-104	2.97	X
TSIX	6.97E-96	2.11E-92	2.91	X
HEPH	2.88E-18	3.11E-15	1.32	X
ARSD	1.20E-08	1.21E-05	1.05	X
MAP7D2	3.47E-08	3.28E-05	1.07	X
KDM5C	7.64E-08	6.80E-05	1.03	X
KDM6A	7.25E-07	6.10E-04	1.06	X
STS	1.13E-06	9.04E-04	1.07	X
PLK1S1	1.00E-05	7.21E-03	1.05	20
CBX7	2.59E-05	1.78E-02	1.05	22
ZFX	3.97E-05	2.61E-02	1.07	X
EIF1AX	8.24E-05	4.99E-02	1.05	X
PKP4	9.46E-05	5.51E-02	1.04	2
FAM117B	1.04E-04	5.86E-02	1.06	2
CEP63	1.09E-04	5.89E-02	1.04	3
SRBD1	1.16E-04	6.03E-02	1.02	2
ZNF254	1.27E-04	6.40E-02	1.09	19
UBA1	1.37E-04	6.68E-02	1.01	X
DHTKD1	1.80E-04	7.51E-02	1.05	10
RABGAP1L	1.82E-04	7.51E-02	1.04	1
CCDC146	1.97E-04	7.66E-02	1.09	7
GAB1	2.17E-04	7.97E-02	1.05	4
HSDL2	2.20E-04	7.97E-02	1.04	9
DPEP2	2.53E-04	8.70E-02	1.04	16
NUP214	2.68E-04	9.03E-02	1.02	9
RPAP3	2.90E-04	9.53E-02	1.04	12
BBX	3.00E-04	9.65E-02	1.03	3
ST6GALNAC2	3.16E-04	9.78E-02	1.03	17
RBP7	3.16E-04	9.78E-02	1.05	1
PARP8	3.30E-04	9.99E-02	1.05	5

Supplemental Table 2

Differentially-Expressed Genes in Blood Samples, including genes on the Sex Chromosomes

More Highly Expressed in Males

Gene	pvalue	FDR	FC	chr
KDM5D	2.39E-107	3.57E-103	0.37	Y
EIF1AY	2.53E-100	1.89E-96	0.37	Y
RPS4Y1	1.04E-97	5.19E-94	0.42	Y
DDX3Y	3.64E-91	1.36E-87	0.61	Y
TTTY10	2.55E-65	5.46E-62	0.65	Y
PRKY	2.31E-64	4.32E-61	0.63	Y
NCRNA00185	3.99E-62	6.63E-59	0.62	Y
UTY	2.56E-52	3.83E-49	0.67	Y
USP9Y	4.75E-48	6.46E-45	0.51	Y
TTTY15	5.09E-29	6.35E-26	0.77	Y
ZFY	1.33E-17	1.24E-14	0.80	Y
STK32B	4.72E-08	2.82E-05	0.93	4
KAL1	5.25E-07	2.91E-04	0.89	X
FRG2C	1.43E-06	6.88E-04	0.95	3
OAF	2.02E-06	9.44E-04	0.96	11
TMSB4Y	5.39E-06	2.44E-03	0.97	Y
ASGR1	1.56E-05	6.86E-03	0.97	17
TSHZ3	2.00E-05	8.10E-03	0.96	19
S1PR3	4.56E-05	1.75E-02	0.95	9
ZCCHC24	9.77E-05	3.31E-02	0.97	10
MGST2	1.29E-04	4.10E-02	0.98	4
ZBED1	1.37E-04	4.26E-02	0.97	X|Y
FXYD6	1.59E-04	4.76E-02	0.96	11
VCAN	1.86E-04	5.29E-02	0.98	5
SPG21	1.91E-04	5.29E-02	0.99	15
PTGDS	1.90E-04	5.29E-02	0.94	9
RASSF4	1.99E-04	5.30E-02	0.98	10
TMEM9	2.07E-04	5.33E-02	0.98	1
HPSE	2.22E-04	5.53E-02	0.97	4
GPER	2.19E-04	5.53E-02	0.97	7
CATSPER1	2.30E-04	5.63E-02	0.96	11
FAM198B	2.37E-04	5.72E-02	0.94	4
MGST1	2.51E-04	5.96E-02	0.97	12
CARD9	2.70E-04	6.12E-02	0.97	9
NUDT16P1	2.78E-04	6.20E-02	0.95	3
ORAI2	4.14E-04	8.48E-02	0.97	7
RPS27L	4.23E-04	8.55E-02	0.96	15
CCNY	4.38E-04	8.73E-02	0.99	10
SDHB	4.51E-04	8.87E-02	0.99	1
RAB32	4.81E-04	9.12E-02	0.98	6
ENSA	4.79E-04	9.12E-02	0.99	1
DACH1	5.33E-04	9.61E-02	0.96	13
GPR162	5.30E-04	9.61E-02	0.96	12
CD63	5.14E-04	9.61E-02	0.99	12

More Highly Expressed in Females

Gene	pvalue	FDR	FC	chr
TSIX	3.74E-84	1.12E-80	3.09	X
XIST	5.06E-79	1.26E-75	3.09	X
MAP7D2	1.21E-21	1.39E-18	1.13	X
KDM5C	1.64E-19	1.75E-16	1.04	X
HEPH	7.33E-19	7.31E-16	1.18	X
PRKX	5.15E-16	4.53E-13	1.07	X
SEPT6	1.77E-14	1.47E-11	1.03	X
DDX3X	3.84E-12	3.03E-09	1.04	X
ZRSR2	5.21E-10	3.90E-07	1.04	X
CA5B	4.99E-09	3.55E-06	1.05	X
KDM6A	7.20E-09	4.68E-06	1.07	X
ZFX	7.08E-09	4.68E-06	1.06	X
ARSD	2.48E-08	1.55E-05	1.03	X
EIF2S3	4.66E-07	2.68E-04	1.01	X
GEMIN8	6.34E-07	3.38E-04	1.03	X
PNPLA4	6.91E-07	3.56E-04	1.07	X
SMC1A	1.13E-06	5.61E-04	1.03	X
EIF1AX	1.90E-05	7.89E-03	1.09	X
KDELC1	1.89E-05	7.89E-03	1.03	13
CD96	2.95E-05	1.16E-02	1.03	3
NFX1	8.37E-05	3.06E-02	1.02	9
GAL3ST4	8.39E-05	3.06E-02	1.04	7
SP140	9.13E-05	3.25E-02	1.02	2
NKRF	9.42E-05	3.28E-02	1.05	X
CD6	9.97E-05	3.31E-02	1.04	11
PPP1R13L	1.24E-04	4.04E-02	1.06	19
XG	1.48E-04	4.52E-02	1.03	X
DFNB31	1.80E-04	5.28E-02	1.05	9
TRAPPC2	1.95E-04	5.29E-02	1.02	X
DACT1	2.02E-04	5.30E-02	1.11	14
RHOH	2.58E-04	5.96E-02	1.02	4
INE1	2.59E-04	5.96E-02	1.03	X
NLRP2	3.15E-04	6.93E-02	1.08	19
MTOR	3.25E-04	7.04E-02	1.02	1
ASMT	3.30E-04	7.05E-02	1.04	X|Y
DENND5B	3.57E-04	7.53E-02	1.05	12
TMC8	3.97E-04	8.25E-02	1.02	17
ZNF248	4.82E-04	9.12E-02	1.03	10
ABLIM1	5.20E-04	9.61E-02	1.02	10
